# Avian Malaria Deaths in Parrots, Europe

**DOI:** 10.3201/eid1705.101618

**Published:** 2011-05

**Authors:** Philipp Olias, Maria Wegelin, Wolfgang Zenker, Sabrina Freter, Achim D. Gruber, Robert Klopfleisch

**Affiliations:** Author affiliations: Freie Universität, Berlin, Germany (P. Olias, S. Freter, A.D. Gruber, R. Klopfleisch);; IDEXX Diavet Labor, Bäch, Switzerland (M. Wegelin);; Animal Clinic Neuwiesen, Uster, Switzerland (W. Zenker)

**Keywords:** malaria, parasites, bird, cytochrome b, Haemoproteus, Plasmodium, Leucocytozoon, host specificity, Besnoitia, Passeriformes, Psittaciformes, letter

**To the Editor:** Avian malaria is an insect-borne disease induced by a so far unknown number of protozoan blood parasites of the genera *Plasmodium* and *Haemoproteus* (hematozoa) (*1**,**2*). The unintentional introduction of *P. relictum* to the Hawaiian Islands, USA, has had fatal effects for the native bird fauna (*3*). In Europe, asymptomatic blood infections by hematozoa have been regularly observed, with an especially high prevalence in songbirds (*4*). However, numerous outbreaks of fatal protozoan infections have been reported over the past 40 years, mainly among psittacines of Australia that have been kept in aviaries (*5**,**6*). Diagnosis in all these cases was based on histopathologic detection of protozoan cyst-like structures of unexplained origin in the heart and skeletal muscles and, to a lesser extent, in other organs. In most cases, the protozoans were identified as members of the genus *Leucocytozoon* because of their morphologic features. Recent studies suggest that these cases may, in fact, have been infections of *Besnoitia* spp. (Sarcocystidae) or other unknown hematozoa (*5*); however, genetic evidence is lacking.

In August 2010, sudden deaths of parrots were noticed in 2 separate aviaries in northern Germany and Switzerland (Technical AppendixTable). Nine yellow-crowned parakeets (*Cyanoramphus auriceps*), 3 barred parakeets (*Bolborhynchus lineola*), and 2 budgerigars (*Melopsittacus undulatus*) died within 2–5 days after a history of reduced general condition and reduced activity and food intake before death. In addition, 2 budgerigars and 1 barred parakeet in the aviary in Germany showed lethargy and reduced food intake for 2 weeks but fully recovered. About half of the birds were juvenile. No new birds had been introduced into the aviaries during the previous 24 months.

Necropsy and histologic examination of 7 animals with fatal disease showed numerous large cyst-like protozoan structures (size up to 800 µm in diameter; Technical Appendix Figure) in myocardial and skeletal muscles and, to a lesser extent, in the lung and the smooth muscles of the intestinal tract without obvious signs of inflammation. The cyst-like structures had a thick eosinophilic outer wall, were partly compartmented by internal septae, and were filled with many merozoites. Surrounding muscle fibers were degenerated or necrotic and, in some cases, associated with hemorrhage. Blood smears of clinically affected animals screened for ≈5 × 10^5^ cells each did not show parasites. To further characterize the parasites, we carried out a nested PCR and subsequent DNA sequencing as described (*7*). Notably, phylogenetic comparison of 479 bp of the mitochondrial cytochrome b gene derived from protozoan cyst-like structures with known sequences of avian hematozoa found 99%–100% homology of parasites from both outbreaks with the avian malaria parasites (*Haemoproteus* spp.) of European songbirds (Figure). Identical cytochrome b sequences were detected in a yellow-crowned parakeet from Switzerland (CYAUR1), a budgerigar from Germany (MEUND1), and a *Haemoproteus* sp. (TUPHI1) previously found in the blood of a song thrush (*Turdus philomelos*) in Bulgaria. The sequence derived from the barred parakeet (BOLIN1) of the German outbreak was identical with *H. minutus* of the common blackbird (*T. merula*). In fact, different psittacine species of the German outbreak were infected with different *Haemoproteus* spp. Because all affected parrots had been bred in Europe and had no contact to imported birds, these results suggest that infection was the result of previously unknown cross-species transmission of *Haemoproteus* spp. between birds of only distantly related orders (*8**,**9*).

**Figure:**
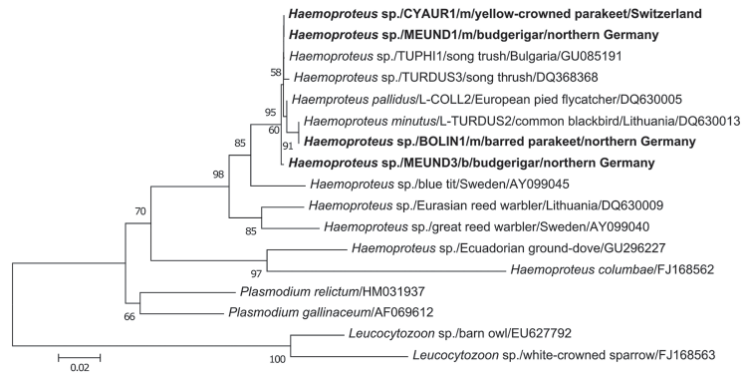
Phylogenetic relationships based on alignment of 479 bp of the cytochrome b gene of *Haemoproteus* spp. isolated from megalomeronts (m) of infected muscles and blood (b) of parrots with related hematozoan parasites in GenBank and the database MalAvi (http://mbio-serv4.mbioekol.lu.se/avianmalaria; *10*). Nucleotide distance values of the maximum likelihood phylogenetic tree were calculated under the HKY substitution model. New sequences of *Haemoproteus* spp. from parrots of this study are shown in **boldface**. Two distinct species of the genus *Leucocytozoon* served as outgroup of the phylogenetic tree. The branch lengths are proportional to the degree of inferred evolutionary change as shown by the scale bar, and the numbers indicate bootstrap values (1,000 replicates). While the cytochrome b sequences CYAUR1, MEUND1, and BOLIN1, respectively, found matching sequences, MEUND3 showed closest sequence similarities with *Haemoproteus* spp. of the lineage COLL2, which depict a wider host breadth among songbirds (http://mbio-serv4.mbioekol.lu.se/avianmalaria). The isolates of *Haemoproteus* spp. from psittacine birds were deposited into GenBank under accession nos. HQ398207–HQ398212.

Blood samples from surviving, asymptomatic animals from the German outbreak were tested cytologically and by nested PCR for the presence of *Haemoproteus* spp. PCR identified *Haemoproteus* sequences in the blood of 3 of 26 psittacines, although parasitic structures were not identifiable in blood smears. Retrieved sequences were identical with that of MEUND1, except for a single-nucleotide polymorphism in 1 sequence (MEUND3; Figure). A latent infection of these animals therefore seems possible and may constitute a potential risk for further horizontal transmission in aviaries by blood-sucking insects such as biting midges (*Culicoides*), the vectors for *Haemoproteus* spp. of passerine birds in Europe (*2*).

In conclusion, we identified the cause of a previously unexplained lethal disease of captive parrots in Europe, induced by numerous large cyst-like megalomeronts in several organs, including the heart. Morphologically, the parasitic structures were strikingly similar to yet undetermined parasites of numerous previous outbreaks (*5**,**6*). Genetically, the parasites had 99%–100% homology to known *Haemoproteus* spp. from wild European songbirds. The avian malaria parasites identified are highly prevalent in the native songbird population but generally do not cause overt disease or death in their natural hosts. In contrast, the cases reported here suggest that these parasites that have adapted to European songbirds may cause fatal outbreaks in native psittacines of Australia, New Zealand, and South America that are raised in captivity. These findings also show that preexisting pathogens may be a potential hazard for invading species. Avian malaria should therefore be considered a threat for exotic parrots in Europe until results of further epidemiologic and experimental studies are available. Because many European bird species have been introduced to the native range of the psittacines studied here, a concern has been expressed that these parasites already have become established in these areas and are affecting the natural populations.

## Supplementary Material

Technical Appendix Table depicting complete list of animals in the affected aviaries and an image of the Myocardium of yellow-crowned parakeet that is severely infected with numerous large megalomeronts of Haemoproteus spp. .
