# Supplementary material for: Avian Malaria Deaths in Parrots, Europe
Source: Emerg Infect Dis. 2011 May;17(5):950–2. doi: 10.3201/eid1705.101618 (PMC3338161; doi:10.3201/eid1705.101618)
Supplement: Technical Appendix — Table depicting complete list of animals in the affected aviaries and an image of the Myocardium of yellow-crowned parakeet that is severely infected with numerous large megalomeronts of Haemoproteus spp. . [file 10-1618-Techapp.pdf]

Article DOI: 10.3201/eid1705.101618

Avian Malaria Deaths in Parrots, Europe

Technical Appendix

Technical Appendix Table. Complete list of animals in the affected aviaries, northern Germany and Switzerland, August 2010

Site location (species)	No. animals died/total	Histologic detection of *Haemoproteus* spp.: Gametocytes in erythrocytes	Histologic detection of *Haemoproteus* spp.: Megalomeronts in heart and skeletal muscle†	PCR detection of *Haemoproteus* spp. in blood
Aviary 1 (Germany), 52°16′N, 13°39′E				
Budgeridgar (*Melopsittacus undulatus*)	2/20	0/18	1/1	3/18
Cockatiel (*Nymphicus hollandicus*)	0/6	0/2	ND	0/2
Barred parakeet (*Bolborhynchus lineola*)	3/6	0/5	1/1	1/5
Eastern rosella (*Platycercus eximius*)	0/2	0/2	ND	0/2
Chicken (*Gallus gallus domesticus*)	0/4	0/4	ND	0/4
Aviary 2 (Switzerland) 47°25′N, 8°50′E				
Yellow-crowned parakeet (*Cyanoramphus auriceps*)	9/18	ND	5/5	ND
Cockatiel (*Nymphicus hollandicus*)	0/6	ND	ND	ND
Domestic canary (*Serinus canaria*)	0/15	ND	ND	ND

*ND, not done.
†Confirmed by PCR.

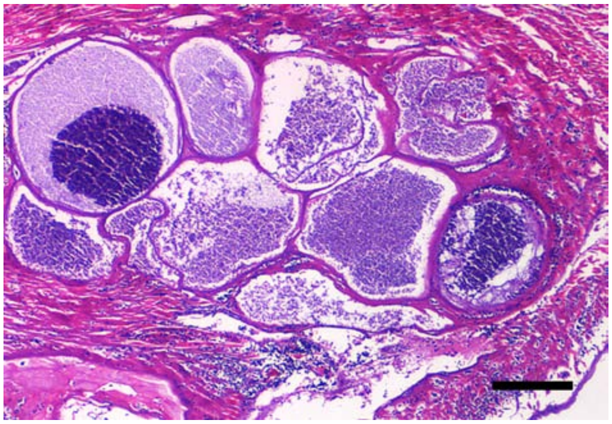

Technical Appendix Figure. Myocardium of yellow-crowned parakeet that is severely infected with numerous large megalomeronts of *Haemoproteus* spp. Note the thick eosinophilic hyaline wall of megalomeront with internal septation and myriads of spherical basophilic merozoites. Surrounding muscle fibers are degenerated, necrotic, and hemorrhagic (hematoxylin and eosin stain). Scale bar = 150 μm.
